# Gray matter volume increase in the retrosplenial/posterior cingulate cortices of blind soccer players

**DOI:** 10.3389/fnins.2025.1462481

**Published:** 2025-04-14

**Authors:** Tomoyo Morita, Eiichi Naito

**Affiliations:** ^1^Center for Information and Neural Networks (CiNet), Advanced ICT Research Institute, National Institute of Information and Communications Technology (NICT), Osaka, Japan; ^2^Graduate School of Frontier Biosciences, Osaka University, Osaka, Japan

**Keywords:** blind soccer, navigation, retrosplenial cortex, gray matter, MRI, hyperadaptation

## Abstract

Individuals typically recognize where they are (localization) and in which direction they are heading (orientation) in a space using vision, and the retrosplenial/posterior cingulate cortices (RSC/PCC), parahippocampal cortex (PHC), and hippocampus (HP) have been shown to play crucial roles for these navigation-related functions. However, there is empirical evidence that top blind soccer players with long-term training can navigate on the court without vision. This study examined the potential changes in gray matter (GM) volume in the RSC/PCC, PHC, and HP in the brains of a leading and other blind soccer players. We collected structural magnetic resonance imaging (MRI) scans from six blind soccer players (including the world’s top player) and eight blind non-soccer players. Using voxel-based morphometry (single-case approach), we compared GM volume in each participant to that of 250 sighted participants (none of whom had ever played blind soccer). The world’s top blind soccer player had a significant increase in GM volume in the bilateral RSC/PCC compared to sighted participants. Two of the other five blind soccer players also showed a GM increase in the left RSC/PCC. However, this increase in GM volume was not observed in blind non-soccer players. Consequently, the probability of a significant GM increase in the RSC/PCC was significantly higher in the blind soccer group than in the blind non-soccer group. In contrast, no between-group differences were observed in the probability of a significant GM volume increase in the PHC and HP. This study, which unveiled the characteristics of the brains of the world’s top blind soccer player and other blind soccer players, demonstrates for the first time that blind soccer training, which requires navigation based on non-visual cues, may enlarge the human RSC/PCC. Moreover, the findings promote our understanding of the brains of visually-impaired persons playing blind soccer.

## 1 Introduction

Vision is the dominant sense humans use for navigation (Ekstrom, 2015). Visual information enables people to instantly perceive their surroundings in a vista space, which can be perceived from one vantage point by turning one’s head (Montello, 1993). Using visual cues as landmarks, people can easily determine where they are (localization) and the direction in which they are heading (orientation), enabling appropriate navigation (Epstein and Vass, 2014). For example, when sighted people play soccer or futsal, they can instantly recognize the spatial locations of the ball, goals, teammates, and opponents, and also recognize where they are and in which direction they are heading on the court, allowing them to navigate themselves to appropriate positions. On the other hand, without visual information, one can easily imagine how difficult this would be.

However, the human brain occasionally exhibits extraordinary adaptations in the absence of vision. A typical example is observed among top blind soccer players. Blind soccer is a popular parasport for visually impaired athletes; it is a 5-a-side soccer game in which four field players wearing an eye mask play based on the sound of the ball and voice communication. The rules are almost the same as those of futsal, but the players listen to the voices of a “guide” who stands behind the goal telling the field players the position, distance, and angle of the goal, of the “goalkeeper” who is a sighted or low-visioned player, and of the “coach” who stands on a bench beyond the side fence. In addition, they hear the term “Voy!” from other players, which is the word they must articulate when approaching someone with the ball to let them know of their presence and avoid dangerous collisions. Surprisingly, top blind soccer players who have been training for a long time are able to run around on the court, pass the ball to teammates, dodge opponents, and kick the ball into the goal, despite their lack of vision (Blind Football World Cup, 2018). This remarkable performance is presumably related to their superior ability to accurately localize and orient themselves on the court, relying not on visual information, but on non-visual information such as auditory (sound and voice) and bodily (e.g., vestibular and proprioceptive) information. There are other blind sports that involve navigation, such as track and field; however, blind soccer is the only sport that involves a type of navigation in which one’s position and destination change from moment to moment but are not navigated by accompanying persons. Therefore, spatial information about one’s location on the court and direction is essential for successful blind soccer, and the brains of blind players must constantly update and maintain this information when playing blind soccer. We hypothesized that years of blind soccer experience would train the ability to localize and orient oneself without vision, which is rarely trained in everyday activities of sighted individuals.

It is now well established that in experts (e.g., athletes and musicians) with specific training over a long period, gray matter (GM) volume increases in brain regions that repeatedly process information related to the content of the training (Maguire et al., 2000; Gaser and Schlaug, 2003; Draganski et al., 2004; Kanai and Rees, 2011; Thomas and Baker, 2013; Gerber et al., 2014). Hence, we hypothesized that years of blind soccer training would increase GM volume in brain regions that play central roles in localizing and orienting oneself in a space without vision.

Meta-analyses of functional neuroimaging studies on navigation in sighted individuals have consistently reported activation in two regions (Spreng et al., 2009; Boccia et al., 2014; Cona and Scarpazza, 2019; Li et al., 2021; Baumann and Mattingley, 2021): the parahippocampal cortex (PHC) and the medial parietal region immediately behind the splenium, which is the caudal part of the corpus callosum. The latter anatomically corresponds to the ventral posterior cingulate cortex (PCC); however, it is conventionally referred to as the retrosplenial cortex (RSC; Foster et al., 2023). Therefore, in this study, we refer to this section as RSC/PCC. Both regions are more active when viewing scenes (e.g., landscapes, cityscapes, and rooms) than when viewing objects (e.g., faces, bodies, artifacts; Epstein and Kanwisher, 1998; Epstein et al., 2003; Downing et al., 2006).

However, a series of studies by Epstein and colleagues have suggested that the PHC and RSC/PCC play different roles in navigation (Epstein et al., 2007; Epstein and Higgins, 2007; Epstein, 2008; Epstein and Vass, 2014). They argued that the RSC/PCC is centrally involved in determining one’s location and orientation in a broader spatial environment, whereas the PHC is primarily concerned with analyzing the local visual scene. This functional difference is supported by neuropsychological findings. When the PHC is damaged, patients cannot identify scenes, although their visual perception remains intact (Pallis, 1955; Landis et al., 1986; Epstein et al., 2001). On the other hand, when the RSC/PCC is damaged, patients can identify the scene or location they are looking at, but cannot use this information to localize and orient themselves (Takahashi et al., 1997; Suzuki et al., 1998; Katayama et al., 1999; Ino et al., 2007; Osawa et al., 2008; Spreng et al., 2009). Navigational functions have mainly been investigated under normal vision in humans. However, considering the aforementioned empirical evidence that blind soccer players likely have the ability to localize and orient themselves without vision, navigation in the absence of vision must be examined. Animal studies have shown that the inactivation of the RSC in rats trained during a maze task in light causes behavioral impairments during the task in darkness rather than in normal light (Cooper and Mizumori, 1999; Cooper et al., 2001). This suggests the importance of the mammalian RSC for navigation without vision. If the human RSC/PCC also plays a crucial role in localization and orientation based on non-visual cues and if it is used frequently in blind soccer training, the GM volume of the RSC/PCC should increase in blind soccer players.

Regarding the PHC, it has been shown that it not only responds when scenes are visible, but also when the scene or navigation is imagined in mind with eyes closed (Ghaem et al., 1997; O’Craven and Kanwisher, 2000; Ino et al., 2002; Rosenbaum et al., 2004). Visually impaired individuals cannot utilize visual information; however, if blind soccer players imagine the scenes of playing blind soccer, as some of them report (see Discussion 4.2), we might expect an increase in the GM volume of their PHC.

In addition to the PHC and RSC/PCC, another brain region that may be involved in identifying one’s location is the hippocampus (HP). This idea originated from a study by O’Keefe and Nadel (1978), in which place cells found in the HP of rodents fired when the animals were in a specific location. It has been reported that the GM volume of the HP was increased in London taxi drivers (Maguire et al., 2000; Woollett and Maguire, 2011) and that HP size is associated with the accuracy of the acquired spatial representation of a campus when students get acquainted with it (Schinazi et al., 2013). This suggests that the human HP is involved in the formation of mental representations of space in an allocentric format (i.e., cognitive maps). Visually impaired individuals have a larger hippocampal volume, especially on the right side, than sighted individuals (Fortin et al., 2008; Leporé et al., 2009), potentially because visually impaired individuals have to generate cognitive maps to compensate for not being able to see actual scenes or maps and to frequently access these maps for navigation. Therefore, the GM volume of the HP might be increased in blind soccer players, if their brains develop allocentric cognitive maps of the court and frequently access them during blind soccer training.

In the present study, we collected structural magnetic resonance imaging (MRI) scans from a world-class blind soccer player who had been playing blind soccer for 17 years and had won multiple world championships. Using voxel-based morphometry (VBM; single-case approach, Scarpazza et al., 2016), we first examined the GM volume increases in the RSC/PCC, PHC, and HP of this top player and compared them to those of 250 sighted individuals. Next, to examine whether the expected GM volume increase in these regions also occurs in other blind soccer players, we performed a single-case VBM analysis on five other blind soccer players. Finally, to investigate whether the expected GM volume increase was caused by blind soccer training, we performed the same analysis on eight visually impaired individuals without blind soccer experience.

## 2 Materials and methods

### 2.1 Participants

Fourteen visually impaired men (age range: 22–42 years) participated in this study, including six persons who had played blind soccer (BS group; mean age, 31.3 ± 6.0 years) and eight without blind soccer experience (BNS group; mean age, 33.3 ± 7.7 years). One participant (BS1) in the BS group was the world’s top blind soccer player, aged 26 years. The participant lost his sight at the age of 8 years and had been playing blind soccer since the age of 10 years. He was a member of the Brazilian national blind soccer team and competed in four consecutive Paralympic Games, contributing to Brazil’s gold medal victories at each event. Four other members of the BS group were current or past members of the Japanese national blind soccer team (BS2, BS3, BS4, and BS5). The remaining participant was not a member of a national team, but had many years of blind soccer experience (BS6). Details of the blind participants, including their clinical features, are summarized in Table 1.

**Table 1:** Visually impaired participants: summary of their clinical features and blind soccer experience.

	Age (y)	Sex	Handedness score	Onset of total blindness (y.o.)	Duration of total blindness (y)	Cause of blindness	Starting age of blind soccer (y.o.)	Duration of playing blind soccer (y)
Blind soccer (BS) group								
BS1	26	M	100	8	19	Retinal detachment	10	17
BS2	26	M	67	23	4	Uveitis	18	9
BS3	38	M	−6	20	19	Retinitis pigmentosa	25	14
BS4	30	M	20	19	12	Microphthalmia + Coloboma	25	6
BS5	41	M	100	23	19	Congenital glaucoma	30	12
BS6	27	M	89	0	28	Unknown	20	8
Blind non-soccer (BNS) group								
BNS1	29	M	6	0	30	Unknown	N/A	0
BNS2	41	M	−50	27	15	Congenital glaucoma + Retinal detachment	N/A	0
BNS3	39	M	76	1	39	Pediatric cancer in optic nerve	N/A	0
BNS4	32	M	89	0	33	Cataracts	N/A	0
BNS5	22	M	100	5	18	Retinal detachment	N/A	0
BNS6	22	M	79	14	9	Congenital chorioretinal atrophy	N/A	0
BNS7	39	M	100	17	23	Uveitis + Glaucoma	N/A	0
BNS8	42	M	100	15	28	Congenital coats disease + Retinal detachment	N/A	0

Handedness scores (from −100 to 100) are determined according to the short version of the Edinburgh test. Positive values indicate relative right-handedness and negative values indicate relative left-handedness. N/A, not applicable; y, years; y.o., years old.

In total, 250 sighted men without experience in blind soccer were also included in this study (sighted group; mean age, 24.1 ± 5.4 years; range, 21–54 years). We obtained datasets from 250 sighted male participants, which included their handedness scores and T1-weighted magnetization-prepared rapid gradient echo (MPRAGE) images taken with the same scanning parameters using the same MRI scanner as that used for the visually impaired participants. These data were not only collected for the current study, but also for several other studies by our group. The age range of the sighted participants was similar to that of the visually impaired participants, although the mean age of the sighted participants was slightly lower. A total of 236 of the 250 sighted participants were asked whether they had trained in sports continuously for more than 1 year in the past, and if so, what kind of sports they had played and how many years they had been playing the sport. Seventy-six of the 236 participants had at least 1 year of soccer experience, and 53 of them had more than 4 years of soccer experience. None of the participants had any history of psychiatric disorders. Handedness was confirmed using the Edinburgh Handedness Inventory (Oldfield, 1971). The mean handedness scores were 61.6 (range: −6 to 100) in the BS group, 62.6 (range: −50 to 100) in the BNS group, and 71.1 (range: −100 to 100) in the sighted group.

The study protocol was approved by the Ethics Committee of the National Institute of Information and Communications Technology and the MRI Safety Committee of the Center for Information and Neural Networks (CiNet; no. 2003260010). The details of the study were explained to the participants before the start of the experiment and they provided written informed consent. In cases where the visually impaired participants could not provide signatures, they orally confirmed their willingness to participate in the experiment and either their guardians, personal attendants, or an experimenter signed the consent form on behalf of the participants in their presence. This study was conducted in accordance with the principles of the Declaration of Helsinki (1975).

### 2.2 MRI acquisition

For each participant, a T1-weighted MPRAGE image was acquired using a 3.0-Tesla MRI scanner (Trio Tim; SIEMENS, Germany) and a 32-channel array coil. Imaging parameters were as follows: repetition time, 1,900 ms; echo time, 2.48 ms; flip angle, 9°; field of view, 256 × 256 mm^2^; matrix size, 256 × 256 pixels; slice thickness, 1.0 mm; voxel size, 1 × 1 × 1 mm^3^; and contiguous transverse slices, 208.

### 2.3 Preprocessing of MRI data

VBM analysis was performed to examine the change in GM volume in each visually impaired participant compared to that in sighted participants (single-case VBM). First, we visually inspected the T1-weighted structural images of all participants and confirmed the absence of observable structural abnormalities and motion artifacts. Data were analyzed using Statistical Parametric Mapping (SPM 12; Wellcome Centre for Human Neuroimaging, London, UK) running MATLAB R2018b (MathWorks, Natick, MA, USA). The following analytical procedures were performed as recommended by Ashburner (2010) and used in our previous study (Morita et al., 2022).

The T1-weighted structural image of each participant was segmented into GM, white matter (WM), cerebrospinal fluid, and non-brain parts based on a tissue probability map provided by SPM. These segmented GM and WM images generated here were then approximated to the tissue probability map using rigid-body transformation, and transformed GM and WM images were generated.

Diffeomorphic Anatomical Registration Through Exponentiated Lie Algebra (DARTEL) processing was performed to generate a DARTEL template, which was subsequently used to accurately align the segmented images across participants. In this process, the average of the transformed GM and WM images across all participants was defined as a template that included the GM and WM. A deformation field was calculated to deform the template into a transformed image for each participant, and the inverse of the deformation field was reapplied to the individual images. This series of operations was performed multiple times to generate a sharply defined DARTEL template.

Thereafter, affine transformation was applied to the DARTEL template to align it with the tissue probability map in the Montreal Neurological Institute (MNI-152) space. The segmented GM image of each participant was then warped nonlinearly to the DARTEL template represented in the MNI space (spatial normalization). Next, the warped image was modulated using the Jacobian determinants of the deformation field to preserve the relative GM volume, even after spatial normalization. Finally, the modulated GM image of each participant was smoothed with an 8-mm full-width-at-half-maximum Gaussian kernel and resampled to a resolution of 1.5 × 1.5 × 1.5 mm voxel size. The smoothed image was used for statistical inference.

### 2.4 Statistical analysis

To compare the GM volume between each visually impaired participant and the 250 sighted individuals, a two-sample non-parametric permutation test (without variance smoothing) was performed using the Statistical non-Parametric Mapping toolbox (version 13.1.09; Nichols and Holmes, 2002). Non-parametric statistics provide a valid alternative to parametric statistics in the context of single-case VBM because they do not rely on the assumptions of normal distribution or equal variance (Scarpazza et al., 2016).

For each statistical comparison, 251 permutations were adopted based on the size (250) of the sighted group. Age, handedness scores, and total brain volume (i.e., the sum of GM and WM volumes) were entered into the design matrix as covariates of no interest to minimize the impact of these variables on the results.

**Figure 1:**
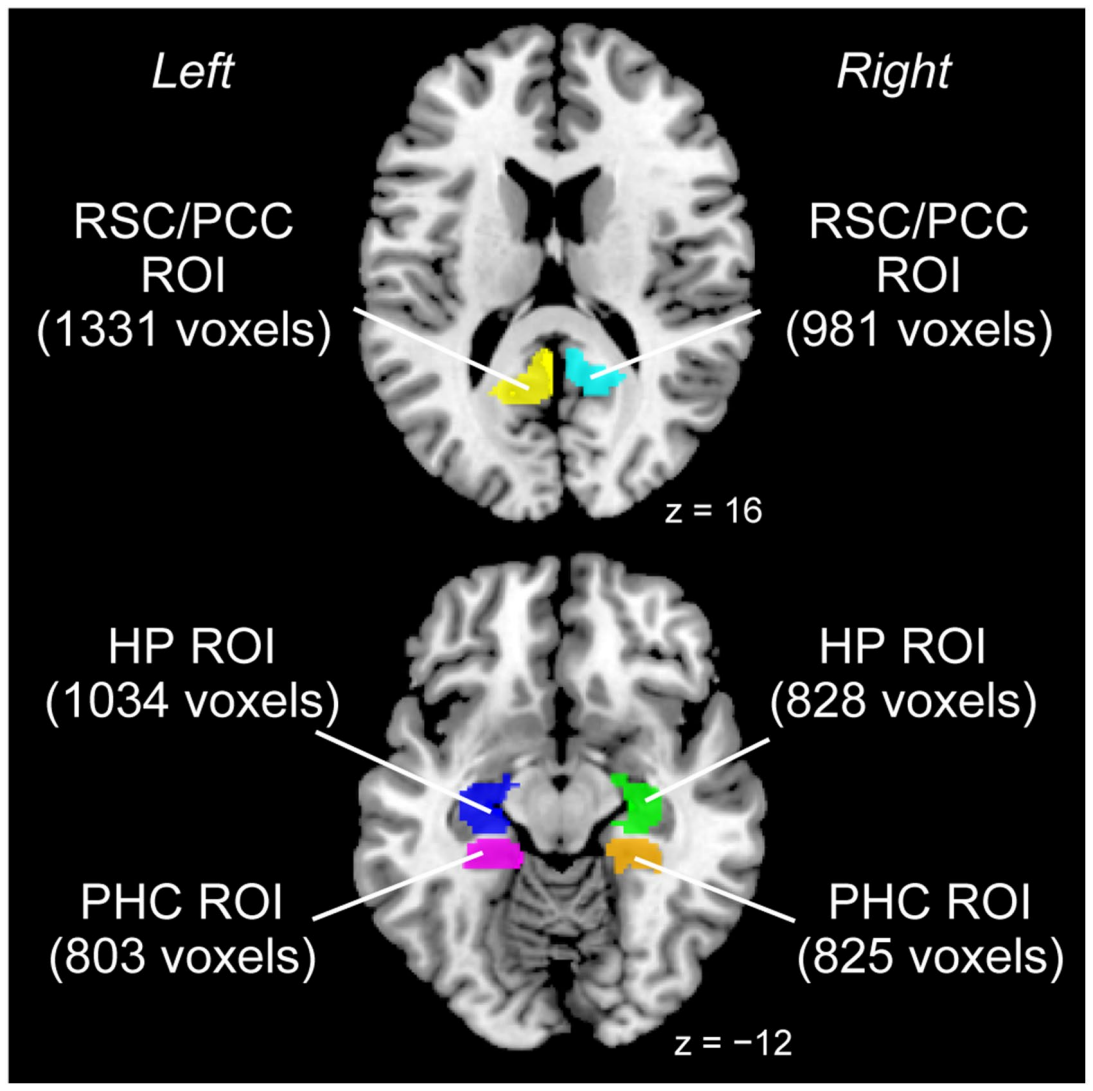
ROIs used for the analysis. Left and right ROIs were prepared for each RSC/PCC (left ROI in yellow, right ROI in cyan), PHC (left ROI in magenta, right ROI in orange), and HP (left ROI in blue, right ROI in green). The size of each ROI is indicated in parentheses (unit: voxels). HP, hippocampus; PCC, posterior cingulate cortex; PHC, parahippocampal cortex; ROI, region of interest; RSC, retrosplenial cortex.

Because we had structural hypotheses for the RSC/PCC, PHC, and HP, we conducted a region-of-interest (ROI) analysis using left and right ROIs for each region (Figure 1). Regarding the RSC/PCC, we initially created a sphere with a 12 mm radius around the peak in the left or right RSC/PCC reported in a previous study preforming a meta-analysis of functional neuroimaging studies on navigation (Cona and Scarpazza, 2019, left RSC/PCC peak coordinates x, y, and z = −12, −50, and 18; right RSC/PCC peak coordinates x, y, and z = 14, −45, and 18). This radius was selected to ensure that the spheres were located anterior to the parieto-occipital sulcus without extending into the occipital cortex. However, these spheres partially overlapped with the corpus callosum, which is a WM region. Therefore, we created a GM mask image from the current data using the SPM Masking Toolbox (Ridgway et al., 2009). Using this image, the left and right RSC/PCC ROIs were defined, excluding the corpus callosum. The left and right RSC/PCC ROIs were 1331 and 981 voxels in size, respectively.

A similar procedure was performed for the PHC. Based on a meta-analysis (Cona and Scarpazza, 2019) that reported a peak only in the right PHC (peak coordinates x, y, and z = 24, −40, and −10), we created a sphere with a 12 mm radius around this peak. For the left PHC, we created a sphere with a 12 mm radius around the right-to-left flipped peak (peak coordinates x, y, and z = −24, −40, and −10) of the right peak to define the putative left PHC. However, these spheres partially overlapped with anatomically defined regions other than the PHC (e.g., the HP). Therefore, the “parahippocampal” region of the Automatic Anatomical Labelling (AAL) Atlas (Tzourio-Mazoyer et al., 2002) was used as the anatomical map of the PHC. Finally, the PHC ROI in each hemisphere was defined by depicting the overlap between the sphere and anatomical map. The left and right PHC ROIs were 803 and 825 voxels in size, respectively.

Regarding the HP, based on a previous study (Leporé et al., 2009) that reported a significant volume increase of the anterior right HP in visually impaired individuals, we created a sphere with a 12 mm radius around the reported right peak (peak coordinates x, y, and z = 25, −17, and −17). For the left HP, a sphere with a 12 mm radius was created around the right-to-left flipped peak of the right peak (peak coordinates x, y, z = −25, −17, −17) to define the putative left HP. Similar to the PHC ROIs, the HP ROI for each hemisphere was defined by depicting the overlap between the sphere and the “hippocampal” region of the AAL Atlas. The left and right HP ROIs were 1034 and 828 voxels in size, respectively.

Within each ROI, we examined the increase in GM volume in each visually impaired participant compared with that in the sighted group. We searched for significant clusters of voxels within each ROI using an extent threshold, *p* < 0.05, Family-Wise Error corrected, for a voxel-cluster image generated at an uncorrected height threshold of *p* < 0.005. We also counted the number of participants in the BS and BNS groups who showed significant clusters within each ROI, and evaluated the between-group differences in this proportion using Fisher’s exact test. In each group, we summed the total number of participants with significant clusters in either the left or right ROI for the left and right RSC/PCC, PHC, and HP ROIs, respectively.

**Figure 2:**
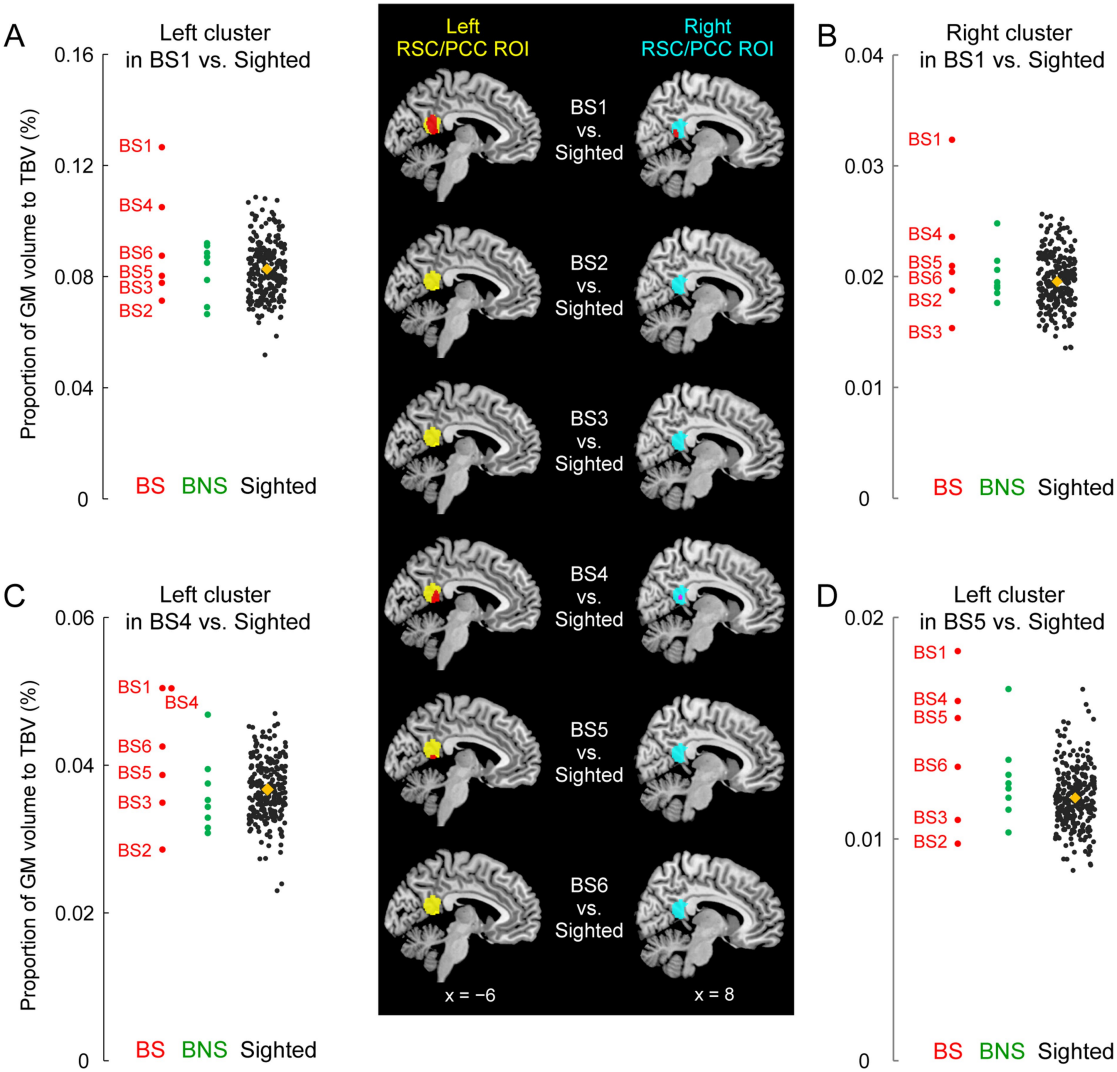
GM volume increase of the RSC/PCC in the blind soccer players. The mapping images on the middle column show results of the comparison between each blind soccer player and the sighted participants (*n* = 250). Red sections indicate significant clusters showing increased volume within the left and right RSC/PCC ROIs (left ROI in yellow, right ROI in cyan). BS1 had a significant cluster in each ROI. BS4 and BS5 had a significant cluster in the left ROI, and BS4 showed a marginally significant cluster in the right ROI (shown in pink, *p* = 0.08). These sections are superimposed on the MNI standard brain, and sagittal slices (*x* = −6 and 8) are shown. Four scatter plot graphs on the left and right columns show the volumes for each individual in each identified cluster (**A**, left cluster in BS1; **B**, right cluster in BS1; **C**, left cluster in BS4; **D**, left cluster in BS5). The vertical axis shows the proportion (%) of the volume of the cluster relative to TBV, corrected for brain size. Each circle represents a participant in the BS (red), the BNS (green), and the sighted (black) group, and orange diamonds represent the means of the sighted group. The plots for the sighted group (including BS4 in panel **C**) are horizontally jittered to avoid over-plotting. BS, blind soccer; BNS, blind non-soccer; GM, gray matter; MNI, Montreal Neurological Institute; PCC, posterior cingulate cortex; ROI, region of interest; RSC, retrosplenial cortex; TBV, total brain volume.

To visualize individual GM volumes in the identified clusters, we extracted the GM volume from the unsmoothed modulated GM image using the SPM get_totals function for each cluster in each participant (Figure 2). Because this value was not corrected for brain size, we corrected it by dividing it by the total brain volume of each participant. These values were plotted strictly for visualization purposes, and no statistical evaluation was performed to avoid the circular assessment issues raised by Kriegeskorte et al. (2009).

## 3 Results

### 3.1 RSC/PCC

Compared to the 250 sighted participants, BS1 showed significant clusters of voxels where the GM volume increased both in the left (peak coordinates x, y, and z = −6, −51, and 14; T = 4.14; 551 voxels) and right (peak coordinates x, y, and z = 14, −45, and 9; T = 3.78; 184 voxels) RSC/PCC ROIs (Figure 2). These were parts of one bilateral RSC/PCC cluster (1034 voxels), the largest cluster in the entire brain, although the size did not reach significance at the whole brain level. A similar increase in GM volume was observed in two other blind soccer players: BS4 and BS5 had significant clusters of voxels in the left RSC/PCC ROI (BS4: peak coordinates x, y, and z = −12, −44, and 8; T = 4.56; 244 voxels, BS5: peak coordinates x, y, and z = −3, −51, and 9; T = 4.20; 82 voxels). In addition, BS4 showed another marginally significant cluster in the right RSC/PCC ROI (peak coordinates x, y, and z = 5, −48, and 15; T = 2.85; 43 voxels; *p* = 0.08). In contrast, none of the blind non-soccer players showed substantial clusters (> 10 voxels) within the RSC/PCC ROIs. Consequently, the probability of significant clusters within the left and right RSC/PCC ROIs (4/12) was significantly higher in the BS group than that (0/16) in the BNS group (Supplementary Table 1, Fisher’s exact test, *p* = 0.02). When we checked the individual GM volumes in each identified cluster, BS1 showed the largest value among all participants, deviating from the distribution of the sighted participants (Figures 2A–D). The left and right RSC/PCC identified as significantly larger in BS1, as compared with the sighted group, were approximately 1.5 times larger than the average size in the sighted group (orange diamonds in Figures 2A,B).

### 3.2 PHC

BS1 did not show significant clusters within the left or right PHC ROIs. Only BS4 showed a significant cluster in the left PHC ROI (peak coordinates x, y, and z = −20, −30, and −8; T = 3.84; 72 voxels; not shown). In the BNS group, none of the participants showed significant clusters, and BNS3 merely showed a marginally significant cluster in the right PHC ROI (peak coordinates x, y, and z = 27, −39, and −3; T = 3.79; 46 voxels). Consequently, the probability of significant clusters within the left and right PHC ROIs (1/12) in the BS group was not significantly different from that (0/16) in the BNS group (Supplementary Table 2; Fisher’s exact test, *p* = 0.43).

### 3.3 HP

BS1 did not show any significant clusters within the left or right PHC ROIs. In the BS group, only BS4 showed significant clusters within the left (peak coordinates x, y, and z = −21, −23, and −11; T = 4.35; 75 voxels) and right (peak coordinates x, y, and z = 26, −23, and −11; T = 4.25; 105 voxels) hippocampal ROIs. Similarly, in the BNS group, only BNS3 showed significant clusters within the left (peak coordinates x, y, and z = −24, −17, and −23; T = 3.16; 90 voxels) and right (peak coordinates x, y, and z = 23, −11, and −17; T = 2.93; 98 voxels) hippocampal ROIs. Consequently, the probability of significant clusters within the left and right HP ROIs (2/12) in the BS group was not significantly different from that (2/16) in the BNS group (Supplementary Table 3; Fisher’s exact test, *p* = 1.00).

## 4 Discussion

### 4.1 General

In this study, GM volume increased in the bilateral RSC/PCC in the world’s top blind soccer player (BS1) and in the left RSC/PCC of two other blind soccer players, although such a significant increase was not observed in blind non-soccer players. Consequently, the BS group had a significantly higher probability of a significant GM increase in RSC/PCC than the BNS group. On the other hand, no between-group differences were observed in the GM volumes of the PHC and HP. We also confirmed the absence of GM increases in the RSC/PCC in sighted participants with regular soccer experience (see Supplementary materials and Supplementary Figure 1), which is consistent with a previous report (Li et al., 2024). This denies the possibility that the present GM increase in the RSC/PCC was likely due to general soccer training. Taken together, these results suggest that blind soccer training, which requires the ability to localize and orient oneself in a space without vision, may increase human RSC/PCC volume and imply that the RSC/PCC plays a central role in these functions.

This was a single-case VBM study of blind individuals. We adopted this approach rather than a group analysis because (1) it allowed us to depict distinct features of GM volume at the individual level when compared to the control group and (2) the sample size in this study was small (six individuals in the BS group and eight in the BNS group); in such cases, the mean in the group could be largely affected by outliers. The small number of visually impaired participants is a limitation of this study (see below), and future application of mixed-effects models or Bayesian statistics (Bernal-Rusiel et al., 2013; Ziegler et al., 2014) in large-scale MRI studies of blind soccer players may clarify whether the GM volume increase in the RSC/PCC that we found is a group effect in general blind soccer population or an individual characteristic which can be observed in blind soccer players. Despite the limitation, our study provides novel evidence that some blind soccer players, represented by BS1, show an extraordinary increase in GM volume in the RSC/PCC. This extraordinary GM volume increase could be referred to as hyper-adaptation, which is defined as an extreme adaptability of the human brain that is different from normal adaptations often observed in force fields and visuomotor adaptations and is rarely seen in the brains of typically developing individuals (see Morita et al., 2022, 2023). Furthermore, such hyperadaptation can be presumed to be achieved through highly motivated, long-term daily training while overcoming the difficulties of disability (Supplementary Figure 2).

The exact physiological changes underlying this increase in GM volume remain unclear. Axon sprouting, dendritic branching synaptogenesis, neurogenesis, and changes in the glial number and morphology have been suggested as important contributors (Zatorre et al., 2012). Increases in GM volume in one brain region are typically use-dependent and related to the duration (amount) of a particular training (Maguire et al., 2000) and the associated skill level (Gaser and Schlaug, 2003; Gerber et al., 2014; James et al., 2014). The greatest GM volume increase in the RSC/PCC (Figure 2) of BS1 appeared to be consistent with these views because he had the longest period of blind soccer training (17 years, Table 1). However, because no significant increase was observed in BS3, who had the second-longest training period (14 years), and a significant increase was observed in BS4, who had the shortest training period (6 years), the training period might not be the only determining factor. Regarding the onset age of training, only BS1 started blind soccer training at the age of 10 in childhood, whereas the others started in adulthood. Our finding that BS1 showed a remarkable volume increase in the RSC/PCC in both hemispheres is consistent with previous studies, suggesting that long-term training with early onset age of the training (i.e., before puberty) may be advantageous for morphological changes (Morita et al., 2022, 2023; Steele et al., 2013). Future studies with more detailed information on the content of blind soccer training might specify factors that essentially contribute the GM volume increase in the RSC/PCC.

All three blind soccer players with increased GM volume in the RSC/PCC lost their sight later in life (late-onset blindness, LB). However, it is unlikely that the GM volume increase in the RSC/PCC reflects the general characteristics of individuals with LB because no study has ever reported this in LB individuals (Paré et al., 2023).

### 4.2 Roles of RSC/PCC in blind soccer players

The RSC/PCC is composed of two major subregions: granular (Brodmann area [BA]29) and dysgranular (BA30) areas (Vann et al., 2009). Most of the RSC/PCC sections where three blind soccer players showed an increase in GM volume appeared to overlap with BA30, which is a posterior subregion of the RSC/PCC. A study combining diffusion tensor imaging and functional connectivity (Li et al., 2018) showed that the superior parietal lobe (SPL), PHC, and visual cortices, which are the structures involved in navigation (Baumann and Mattingley, 2021; Vann et al., 2009), are anatomically and functionally connected to BA30.

A higher ability to localize and orient oneself without vision is considered an advantage for playing blind soccer because players cannot navigate themselves to the appropriate position without accurate localization and orientation. To achieve accurate localization and orientation, they must rely on auditory cues (sounds and voices) from their surrounding environment, rather than on vision. The RSC may be involved in this processing, because there is evidence that the RSC is recruited for spatial processing by non-visual cues as well as by visual cues (Wolbers et al., 2011). However, it is known that BA30 does not have close anatomical and functional connections with auditory cortices, but rather that the anterior RSC/PCC subregion (BA29) has connections with the auditory cortices (Li et al., 2018). Therefore, it is unlikely that the RSC/PCC, whose GM volume increased, is involved in spatial processing based on auditory information because it is a posterior subregion that does not receive sound information from the auditory cortices; however, the possibility cannot be completely ruled out from the present data.

In addition, blind soccer players must rely more on path integration, in which the brain integrates self-movements over time to update where they are and in which direction they are moving, probably by utilizing vestibular, motor, and proprioceptive information. This egocentric (self-centered) sensory-motor information must remain associated with allocentric (world-centered) information about the spatial map of the court to update spatial locations within the court. Among the above-mentioned cortical regions involved in navigation with vision (Vann et al., 2009), egocentric information has been proposed to be processed in the parietal cortex and allocentric information in the PHC and visual cortices. The RSC/PCC is situated in a region that connects these areas and is, therefore, an appropriate place for the association between egocentric and allocentric information (Vann et al., 2009; Clark et al., 2018). In addition, the RSC/PCC is believed to be involved in this association function (Aguirre and D'Esposito,1999; Byrne et al., 2007; Maguire, 2001). Patients with hemorrhage in the right RSC may have difficulty finding their way with the aid of a map, which requires the transformation and integration of egocentric and allocentric information (Suzuki et al., 1998).

This assumption appears to be supported by the functional connectivity results obtained from our additional functional MRI experiments. Related to the current study, we also measured brain activity using the same scanner while the same 14 visually impaired participants and 16 of the 250 sighted individuals performed an imaginary navigation task [see details in Supplementary materials and Amemiya et al. (2021)]. The task was a mental rehearsal of walking or jogging around a circle with a 2 m radius, which was previously experienced without vision on a different day. Among the visually impaired participants, only BS1 showed marginally significant clusters (*p* = 0.059, corrected) in the early visual cortices (peak coordinates x, y, and z = −8, −84, and 16 in area hOc1[V1]; T = 5.37; 65 voxels) and right SPL (peak coordinates x, y, and z = 14, −52, and 74 in area 5 L; T = 7.17; 115 voxels), in which activity enhanced functional coupling with that in the left RSC/PCC ROI (seed) during the task, compared to the sighted participants (see Supplementary Figure 3). It appears that when BS1 performs mental walking or jogging, the RSC/PCC is likely communicating with the SPL (area 5) with an egocentric framework (Scheperjans et al., 2005, 2008; Naito et al., 2008) and with the visual cortices that innately have an allocentric (retinotopic) framework (Striem-Amit et al., 2015), which corroborates the view that the RSC/PCC is situated in an appropriate place for the association between egocentric and allocentric information. These results allow us to infer that BS1 repeatedly uses the RSC/PCC for the association between egocentric and allocentric information to localize and orient himself on the court.

The enhanced functional coupling between the RSC/PCC and visual cortices in BS1 is worth further discussion. After the functional MRI experiment, BS1 reported that he could mentally visualize the walking or jogging scene during the task, as well as the court scene in which he was playing blind soccer. Therefore, it would be worth studying whether such functional coupling is associated with mental visualization of movement in space.

### 4.3 Parahippocampal and hippocampal cortices

Unlike the RSC/PCC, the PHC did not show a significant difference in volume between the BS and BNS groups. This result suggests a functional difference between these two regions, which is consistent with previous studies (Epstein et al., 2007; Epstein and Higgins, 2007; Epstein, 2008; Epstein and Vass, 2014). It is widely known that the PHC shows stronger activity when viewing scenes than when viewing objects (see Introduction). Considering the accumulated evidence on the PHC, it is presumed to be involved in the analysis of visual scene information. In the brains of visually impaired individuals, the PHC does not process bottom-up visual scene information, because no visual input reaches the brain. Nevertheless, unlike their visual cortices where GM atrophy is observed, no GM atrophy has been reported in their PHC (Noppeney et al., 2005: Pan et al., 2007; Ptito et al., 2008; Leporé et al., 2010; Jiang et al., 2015). Therefore, they might utilize the PHC for information processing other than bottom-up visual scene information; however, this usage may not be specifically required for playing blind soccer.

Similar to the PHC, no significant difference was observed in HP volume between the BS and BNS groups. On the other hand, several previous studies have reported that visually impaired individuals have enlarged hippocampal volumes (especially on the right side) compared to sighted individuals (Fortin et al., 2008; Leporé et al., 2009). Consistent with previous findings, the visually impaired group (*n* = 14) in the present study had a significantly larger GM volume in the right HP than the sighted group (Supplementary materials and Supplementary Figure 4). The larger HP volume at the group level indicates that visually impaired individuals might use the HP more frequently than sighted individuals to generate and access cognitive maps to compensate for the lack of visual information. However, the present results imply that the HP is not specifically used in blind soccer training, where the location of the surroundings (e.g., ball, teammates, and opponents) and destination may change from moment to moment.

### 4.4 Limitations

This study has some limitations. First, caution should be exercised when interpreting findings obtained from single-case VBM, even when compared with a large number of control participants, because single-case VBM analysis may identify the effects of individual characteristics (e.g., lifestyle and personality) that we do not intend to investigate. One should bear in mind the possibility that the presented increase in GM volume was due to other factors unrelated to blind soccer training.

Second, the sample size of this study was small; thus, the findings cannot be generalized to a larger population of blind soccer players and visually impaired persons without blind soccer experience. Based on the current results, it is impossible to determine what percentage of the much larger population of blind soccer players has an RSC/PCC volume increase and whether such an increase is truly absent in the larger population of blind non-soccer players. In addition, this study included only one blind soccer player with early onset blindness (EB), who did not show an increase in RSC/PCC volume; thus, the effect of EB on GM volume increases in the RSC/PCC remains unclear. Further research with a large population of blind soccer players, including both EB and LB soccer players, could clarify the impact of blindness onset on RSC/PCC volume changes. Furthermore, we did not compare our data from blind soccer players with those from other blind athletes (e.g., blind marathon runners). Such comparisons in the future are necessary in order to support our claim that increased GM volume in the RSC/PCC is exclusively associated with blind soccer training.

Finally, there is no behavioral evidence directly showing that blind soccer players have superior navigation abilities without vision. Ideally, functional MRI studies using navigation tasks cued solely by auditory and bodily information without vision might help elucidate the functional role of the RSC/PCC in blind soccer players.

## 5 Conclusion

Vision is the dominant sense that humans use for navigation, and most previous studies have investigated the roles of the RSC/PCC, PHC, and HP in navigation in sighted individuals. According to the empirical evidence that leading blind soccer players with long-term training can navigate themselves on the court without vision, the current study focused on navigation in visually impaired individuals and revealed that the volume of the RSC/PCC was significantly increased in a top blind soccer player and in other blind soccer players compared to sighted individuals, which was not observed in blind non-soccer participants. These findings suggest that blind soccer training may increase the GM volume of the RSC/PCC, which plays a central role in spatial self-localization and orientation without vision. Although the sample size was limited, the current study has elucidated the role of the human RSC by unveiling the neural underpinnings of the outstanding spatial ability of the world’s top blind soccer player and has greatly advanced our understanding of the brains of visually-impaired persons playing blind soccer.

## Data Availability

The original contributions presented in the study are included in the article/Supplementary material, further inquiries can be directed to the corresponding author/s.
